# Buffalo Medical and Surgical Association

**Published:** 1882-11

**Authors:** Clayton M. Daniels

**Affiliations:** Secretary


### Society Reports.

### BUFFALO MEDICAL AND SURGICAL ASSOCIATION.

*Regular Meeting, October 2d, 1882.*

President, Dr. A. R. Davidson, in the Chair. Dr. C. M. Daniels, Secretary.

Present—Drs. Coakley, Mynter, Brecht, Hopkins, Johnson, Burghardt, Hartwig, Dagenais, Dorland, Runner, Moody, Cronyn, Van Peyma, and, by invitation, Drs. Tremaine, Potter, Frank, Fell, Heath, Elwood, Messrs. Webster and Marcellus.

Applications for membership received from Drs. W. S. Tremaine, W. H. Heath and W. W. Potter, who were duly elected.

*Essay*—By Dr. C. C. F. Gay; subject: "An inquiry into the source and cause of secondary or recurrent hemorrhage after ligation of arteries for aneurism." The paper appears in the present number of the BUFFALO MEDICAL AND SURGICAL JOURNAL.

*Discussion*—Dr. Tremaine thanked the essayist for his interesting paper. The subject had always been one of deep interest to practical surgeons. In a certain proportion of cases of ligation of arteries for aneurism, secondary hemorrhage did take place, and it was generally due to ulceration of the coats of the vessel, caused by the ligature. Now, if we can find a ligature that will excite sufficient irritation to induce proliferation of the cells of the intima and resulting newly-formed tissue to occlude the lumen of the vessel, and without producing suppuration and ulceration, hemorrhage could not take place at or near the point at which the ligature was applied; in this respect there was, probably, a great advantage in the antiseptic cat-gut ligature of Mr. Lister. As to whether the internal and middle coats of the artery should be divided, or simply pressure enough exerted by the ligature to close the lumen of the vessel sufficiently long to cause an organizable clot to be formed, had, of late, been much debated among surgeons. Mr. Barwell had introduced, and used with success, a flat, tape-like ligature, prepared from the fresh aorta of the ox, with a view to carry out the latter idea. The question was one that experiment alone could solve. When the ordinary ligature was applied sufficiently tight to cause division of the middle and internal coats, the intima curled up and assisted in the formation of the conical, fibrinous clot, which he believed gave additional security; but examination of morbid specimens had shown that even then this clot did not always form, indeed, he (the speaker) had an opportunity of verifying this in a case of his own, where there was a diaphragm-like occlusion without either proximal or distal clot. Sometimes, where these clots did not form, and there was simply the soft, yielding, newly-formed tissue, the impulse of the column of blood might cause it to give way and the lumen of the vessel to be restored. We must always remember that where there was aneurism we were dealing with vessels either diseased or prone to disease, and the necessary healthy reparative action might not take place. He believed that in general it was safer that the ligature be so applied as to divide the inner and middle coats, but insisted that there was greater safety in the antiseptic ligature, as less liable to produce ulceration through the coats of the vessel, the animal ligature becoming organized. The whole subject of secondary hemorrhage was extremely interesting, and reminded him of his experiences during the war, when it came daily, almost hourly, under his observation.

Dr. Heath had seen many cases of aneurism and of subsequent hemorrhage after operation. Cited cases where greatest care was used and where compresses were of no avail. Considered that the condition of the patient had much to do with the danger to be apprehended.

Dr. Potter considered the subject germane to the point of secondary hemorrhage other than that connected with aneurism. Mentioned instances where it had occurred under his own observation; one especially he called to mind where it took place on the ninth day, after laceration of the perineum, which was finally controlled by a tampon, saturated in an alum solution.

Dr. Mynter thought that nearly all questions had been answered. Personally he considered that the cause of secondary hemorrhage, after ligation for aneurism, was always from atheromatous degeneration and the power of contraction of the vessels consequently being lost. Also, that clots often form, but are not invariably present. As to ligatures, he thought it dangerous to use silk; cat-gut, also, might unravel. In ligating he considered it sufficient to draw just tight enough to allow granulation to progress, complete occlusion being unnecessary.

Dr. Cronyn said: "An inquiry into the source and cause of secondary or recurrent hemorrhage after ligation of arteries for aneurism," being a subject of greatest interest, the Society ought to be grateful to Dr. Gay for his effort, not alone to present the various forms of ligature which have been thought most likely to either cause or prevent the hemorrhage, but to suggest a probable new source of cause, entirely independent of the ligature. For myself, I think that the etiology of the special case will always, more or less, determine, not only the nature of the operation to be performed, but the special value, or necessity, for this or that kind of ligature, when it is recollected that, in a large proportion of cases, there existed, prior to the formation of an aneurism, an endo-arteritis; and that very often a number of aneurisms are found in the same person from this cause. It seems to me, therefore, that, when this diseased condition is known to exist, tape-like ligature would be of greatest value, for obvious reasons; but that, in a healthy vessel, as in traumatic aneurism, the silk usually used for tying arteries, or suture, will answer every purpose. So much for the ligature; but what causes the secondary hemorrhage? Dr. Gay thinks it very probably venous in character many times, if not always. Now there is a good deal of reason to suppose that the vessels engaged in the collateral circulation being required by the *vis a tergo* to dilate more rapidly then they are prepared to do, and by their inability to continue the normal *vis a fronte*, a venous stasis will take place in the neighborhood of the ligatured vessel, and to such a degree as to lead to rupture and hemorrhage. This may be the reason why the hemorrhage is so readily, as a rule, controlled by pressure, and why authors recommend it as the best mode of procedure; and where the author of the paper, from his experience, has found his hypothesis. Be this as it may, it will be well to recollect it when ligating diseased arteries for aneurism.

Dr. Hopkins remarked that it hardly became him as a practitioner of medicine to discuss a purely surgical subject. But referring to the literature upon the subject, said the notion is now upon the ligature itself, losing sight of the fact of interference of force in the limb beyond the point of ligature, venous stasis being always present, seemingly to favor any chemical changes that might occur.

Dr. Gay, in closing the discussion, said more time is required than the limits of the paper would allow to do justice to a subject of this nature. Proof, however, had been furnished that the source of secondary hemorrhage is not always at the place where the ligature is applied. A theory, founded upon the resistance to the destructive and degenerative processes of the arterial tissue, is advanced without attempt at elaboration, to account for source of secondary hemorrhage. The autopsy referred to showed the artery had preserved its cylindrical form two months after it had been divided by the ligature. The experiment cited, of injection of water into the common iliac artery, furnishes a link in the chain of evidence in support of the theory alluded to. When the femoral artery was divided below the ligature, water, instead of returning through the iliac vein, entered and passed out through the femoral artery. How did the water get there? You will answer by saying this is accomplished through the anastomotic system of vessels, hence follows the legitimate conclusion that when the circulation in any large vessel has been, for a time, diverted or obstructed—so soon as the collateral circulation has fulfilled its temporary purpose in maintaining the life of the limb—the blood current will return again to its normal channel. As a proof of this, the fact is referred to of recurrent popliteal aneurism. Pressure is so forcible when the return current occurs as, probably, to rupture a branch of the anastomotic vessels, when the blood forces its way back through the open wound.

*Voluntary Communications*—Dr. Hartwig presented a paper on "Operative reunion of a broken patella after two years; success almost perfect." Mr. Herlan, of this city, a square-built and muscular man, fell, two years ago, breaking his patella transversely. The condition was not recognized by the attending physicians, and the joint merely treated with ointments. After a time he walked with crutches, and finally with a cane. In slippery weather he fell five times during two years, and was each time laid up a few days on account of pain in the knee. His condition annoyed him so much that he requested me to unite his patella operatively, declaring definitely that no appliance would satisfy him, though I duly explained all the risks of the operation. Status—Patient walks with his foot very strongly outwards, bends the knee 110 degrees and cannot stretch it actively, but helps himself with the other leg. Only very insignificant ability to extend the leg during walking. Separation of the fragments over four inches. When the leg is extended, I can move the upper fragment one-half inch downwards. Operation 28th of February, 1882, Dr. Heath, Marine Surgeon, Dr. Packwood and Dr. Frank, of this city, assisting. Four per cent. carbolic acid spray clogged, after a while, and was substituted by repeated syringing with two per cent. solution, Lister's protective silk and ten per cent. salicyl cotton for dressing. Narcosis with chloroform; incision on the outside. In the joint many adhesive bands and a quantity of sub-patellar fat cut away. Edges of patella pared with a strong knife. Two holes drilled in each piece, obliquely towards the edge, not touching the joint surface of the patella. I used for drilling tooth-drills, which are very unsuitable, because stouter in the shank than in the drill-head. Thus, one head breaking off, I left it in the patella and bored another hole adjoining. Now, strong hooks were inserted into the tendon of the quadriceps and we tried to pull the patella down, but that was impossible and only strong jerking of the quadriceps the consequence. Thus I was compelled to cut the quadriceps across, while my assistants pulled on the tendon. I had to cut down to the bone and on the inside, near the femoral artery, before the patella yielded sufficiently. The inter-muscular tendinous extensions formed the resistance. Ligating the muscular branches, drawing strongest double silver wires through the patellar pieces, two single wires through the adjoining fasciform tissue, cutting a hole on each side of the joint for drainage tubes and sewing the knee wound with cat-gut, finished the operation. Posterior splint along the whole leg. Volkmann's canvass with the center hole to lie upon. Upper wound left open, but covered with modified Lister's dressing. The shock was considerable, but in the evening reaction set in and the temperature rose to 100°; on the next day to 101; third day, 100½; fourth day, 100; after a few days the temperature went down to normal, rising occasionally ½ to 1 degree. This was clearly a perfectly aseptic fever, and insignificant in itself.

Proof—The patient had splendid appetite from the third day. Primary union of knee wound; not a drop of matter formed. On the fifth day one, on the eighth the other drain was removed. Now the open wound began to granulate, and after four or five weeks the wound was considerably smaller, and epithelium began to cover the surface, while the patient showed, already, some ability to move the tibia. Had I left it as it was, a very susceptible deep scar would have formed. Therefore, and to shorten the process, I scraped the surface of the granulations off a little, lifted, with the shears, the skin all around for ½ to 1 inch, and united the edges of the skin by sutures, leaving in the corner tubes for drainage. This *sit venia verbo* tertiary union took place almost through the whole length, and the space underneath filled up a little yet. Patient got up, though a few points were oozing a little, and walked, after eight weeks, with crutches. At present he does not require even a cane. Meanwhile, the skin has started to ulcerate at a point where, not unlikely, one of the wires in the fasciform extension, on the side of the patella, may come out yet. It will certainly do no harm. In the sitting posture the angle is 45°. This is as much as the patient can bend the knee spontaneously. Slight œdema of the leg prevailed a long while, of course owing to the transverse scar. As far as the patient can bend his leg, he has complete power. Probably more bending would have been possible, if I had instituted early passive motion, but the upper wound did not admit of doing so and I risked a too stiff joint rather than to incur the risk of inflammation. Epicrisis—This is an additional triumph of Listerism. Truly, there are already cases on record. Agnew gives one after nine weeks, and Kœnig mentions one of his own as successful; but while these are all the cases I know of, some unsuccessful ones may not have been published, or they have escaped my search. Gross, Holmes, Hamilton, Bardcleben, Billroth, and other standard works are, so far as accessible to me in Buffalo, altogether silent about the neglected fracture of the patella. For recent cases this proceeding would certainly be easy, and, with perfect Listerism, without danger. Yet, according to my experience, Malgaignes hooks are sufficient, and, without Listerism, safe. Perhaps withdrawing the blood with Dieulafois' aspirator would advantageously precede. I do not think that Lund's proceeding (*Am. Journ.*, April, 1882) is better. For perfectly loose pieces in the joint, opening, seemingly, may be *the* thing anyway (with removal of the pieces); but for neglected cases like ours the injury is, beyond comparison, more extensive. In this case the inside of the joint was hardly recognizable as such. Here I think the patient should be provided with mechanical support and the surgeon should not undertake to operate before he gives his patient a full and truthful explanation of the dangers incurred.

Dr. Mynter considered the result of Dr. Hartwig's operation certainly a brilliant one. Also, raised the question of non-union of bone, and said he thought it was from bleeding that continued so long that the muscles had time to firmly contract, and that on this account operations were often failures.

Dr. Heath, who was present at the time of Dr. Hartwig's operation, said the surroundings at the time were not at all favorable. He thought that the swelling was due to serum and not to blood as put forth by Dr. Hartwig.

Dr. Hartwig further detailed his course in the matter by explaining the necessities and urgency of the case, which were evidently sufficient to warrant such an unusual surgical procedure, as the result readily proved. He also warmly recommended antiseptic precautions.

Referring to the opening of joints, Dr. Cronyn remarked that it was not an extremely unusual thing for good results to follow, and mentioned a case where he opened the knee of a lady twenty-three years ago. Used compound tinct. of benzoin for dressing, and the results were all that could be desired.

Adjourned.

Clayton M. Daniels, *Secretary.*

